# An Accidental Discovery of Twenty 312-Size Zinc and Mercury-Containing Disk Batteries in a Sexagenarian Male

**DOI:** 10.7759/cureus.83075

**Published:** 2025-04-27

**Authors:** Ambika Kapil, Sophie Schuelke, Sohair Angly, Maray Rocher, Sahar S Abdelmoneim

**Affiliations:** 1 Osteopathic Medicine, Nova Southeastern University Dr. Kiran C. Patel College of Osteopathic Medicine, Fort Lauderdale, USA; 2 Internal Medicine, Larkin Community Hospital, Miami, USA; 3 Internal Medicine, Larkin Community Hospital Palm Springs Campus, Hialeah, USA; 4 General Internal Medicine, Larkin Community Hospital Palm Springs Campus, Hialeah, USA; 5 General Internal Medicine/Cardiovascular Medicine, Assiut University Hospital, Assiut, EGY

**Keywords:** adult, battery ingestion, colonoscopy retrieval, foreign body ingestion, lower gastrointestinal tract

## Abstract

Foreign body ingestion, while commonly observed in children, also occurs in elderly individuals. Button batteries pose a significant risk that can lead to rapid tissue damage. Herein, we present a case of a 61-year-old Hispanic male with a past medical history of coronary atherosclerosis, hypertension, asthma, and hyperlipidemia, who initially presented with shortness of breath and bilateral lower limb swelling. However, a passive complaint of constipation prompted further imaging, which unexpectedly revealed multiple foreign bodies in the gastrointestinal tract. A colonoscopy was performed, retrieving 14 button batteries, with additional batteries eliminated through a bowel regimen. This case highlights the need for a high index of suspicion for foreign body ingestion in elderly patients, even in the absence of classic symptoms. It illustrates the importance of thorough history-taking and early imaging in patients with unexplained gastrointestinal symptoms, particularly when initial diagnoses do not fully explain the clinical picture. Given the growing aging population and increased risk of accidental ingestion, further research is needed to establish optimal management strategies and prevent severe complications in similar cases.

## Introduction

Elderly individuals face unique risks when ingesting button batteries, making this population particularly vulnerable to severe complications. While ingestion of disk batteries most commonly occurs in children, a second age peak occurs in elderly patients, with those aged 60 to 89 years accounting for 4% to 16% of all reported incidents [1]. Unlike pediatric cases, button battery ingestion in elderly individuals is typically accidental and driven by age-related factors such as cognitive impairment, neurological disorders, and swallowing difficulties (dysphagia). Additionally, polypharmacy and impaired sensory function may contribute to unintentional ingestion, as some medications cause dry mouth or reduced awareness of foreign objects in the oropharynx.

Once ingested, button batteries pose a serious risk, as they generate an external electric current when lodged in the esophagus, rapidly causing tissue damage. The release of alkaline substances from the battery further exacerbates mucosal injury, causing liquefactive necrosis within hours [2]. In addition to local tissue destruction, the heavy metals and toxic compounds within the battery can contribute to systemic toxicity if leakage occurs, increasing the potential for severe complications. Esophageal perforation, tracheoesophageal fistulas, and mediastinitis are among the most severe complications, with a higher morbidity and mortality rate in elderly individuals due to delayed recognition and comorbidities [3].

Further complicating diagnosis, affected patients often present with nonspecific symptoms that can mimic common age-related conditions or viral respiratory and gastrointestinal illnesses, such as nausea, cough, dysphagia, odynophagia, or hematemesis [4]. Hence, early radiographic localization is crucial in identifying the battery’s position, diameter, and composition, as these factors determine the urgency of intervention [5]. Esophageal impaction necessitates emergent endoscopic removal to prevent severe injury, whereas batteries that have passed into the stomach may be managed with expectant supportive care, depending on symptomatology and progression on serial imaging [4]. The decision to proceed with immediate retrieval versus observation depends on clinical presentation, patient risk factors, and the potential for obstruction or mucosal injury [6].

The patient was informed that data concerning the case would be submitted for publication, and he provided informed consent.

## Case presentation

We present a 61-year-old Hispanic male with a past medical history of coronary atherosclerosis, hypertension, asthma, and hyperlipidemia, who initially presented to the emergency department with shortness of breath at rest and increasingly bilateral lower limb swelling over the past two weeks. His symptoms were associated with generalized malaise and bilateral lower abdominal pain. He denied any fever or chills. Home medications included arformoterol (15 mcg/2 mL) inhaled twice daily, aspirin (81 mg) one tablet daily, atorvastatin (80 mg) one tablet at bedtime, buspirone (5 mg) one tablet twice daily, metoprolol (50 mg) twice a day, prednisone (10 mg) daily, and ibuprofen (400 mg) as needed.

**Figure 1:**
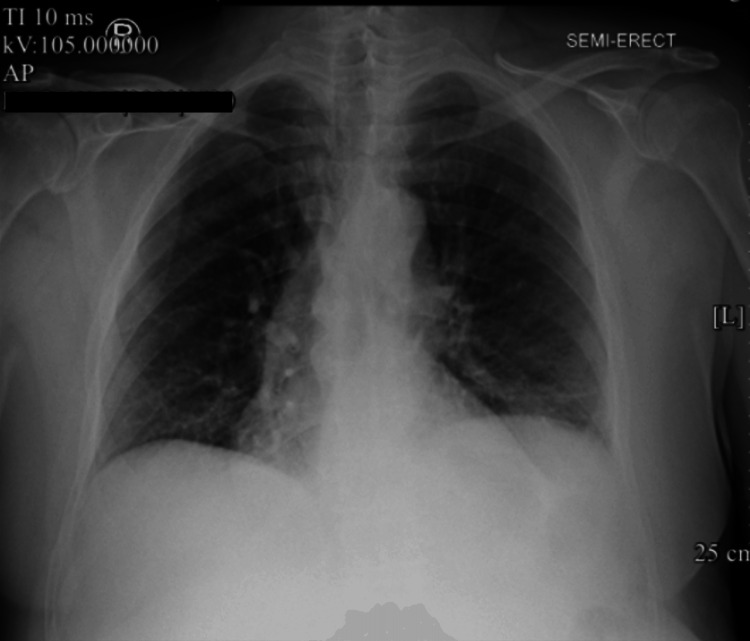
Chest X-ray on admission showing no focal pulmonary consolidations or pleural effusions.

**Figure 2:**
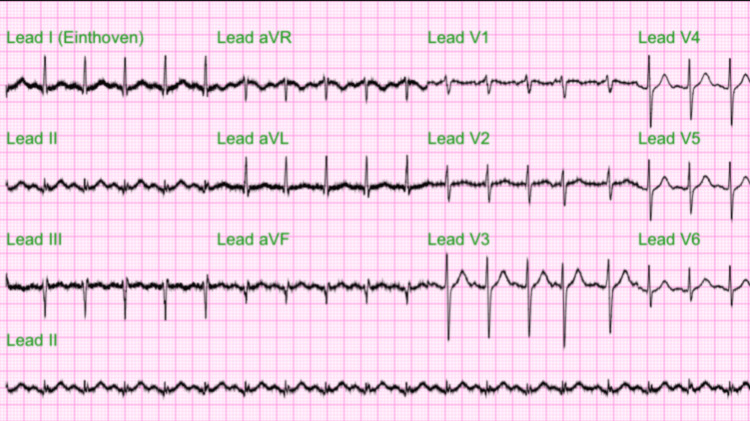
Baseline electrocardiogram (EKG). Shortened R-R interval with unremarkable findings in the P-waves, QRS complex, T-waves, and S-T segments demonstrating sinus tachycardia. Heart rate was 117 bpm.

Vital signs at admission were blood pressure at 143/92 mmHg, heart rate of 125 beats per minute, and oxygen saturation of 96% on a 2-liter nasal cannula. Physical examination was positive for a short systolic murmur over the apex, bilateral lower limb pitting edema up to the knees, bilateral diffuse wheezing, and bilateral abdominal lower quadrant tenderness on deep palpation. Chest X-ray on admission was unremarkable (Figure 1). The electrocardiogram (EKG) showed sinus tachycardia (Figure 2). The transthoracic echocardiogram (TTE) showed mild concentric left ventricular hypertrophy with grade-II diastolic dysfunction, preserved left ventricular ejection fraction (LVEF = 55-60%), mild mitral and tricuspid regurgitation, and an elevated systolic pulmonary artery pressure of 35 mmHg (reference range: 18-25 mmHg). The patient tested positive for influenza A. The patient was negative for influenza B and COVID-19. Complete blood count, complete metabolic panel, urine drug screen, and urine analysis were unremarkable. Troponin was negative, less than 0.01 ng/mL (reference range: <0.04 ng/mL). Pro-brain natriuretic peptide (pro-BNP) was mildly elevated at 208 pg/mL (reference range: <400 pg/mL). The remainder of the lab values during admission are shown in Table 1.

**Table 1:** Laboratory values during admission.

Test	Admission	Reference range
Sodium (Na)	135	135–145 mmol/L
Potassium (K+)	3.9	3.5–5.0 mmol/L
Chloride (Cl)	101	98–107 mmol/L
Bicarbonate (HCO3)	26	22–28 mmol/L
Blood urea nitrogen (BUN)	22	7–20 mg/dL
Creatinine	0.71	0.6–1.2 mg/dL
Glucose	87	70–99 mg/dL (fasting)
White blood cells	9.1	4.5–11.0 × 10³/μL
Neutrophil	81.3	30–75%
Lymphocyte	9.8	20–45%
Monocyte	7.4	0–10%
Eosinophil	0.4	0–6%
Mean corpuscular volume (MCV)	88.2	80–96 fL (female)
Hemoglobin	12.3	12.0–15.5 g/dL
Hematocrit	36.8	36.0–44.0%
Platelet	146	150–400 × 10³/μL
Sedimentation rate	21	0–21 mm/hr
Calcium	8.3	8.5–10.5 mg/dL
Albumin	3.4	3.5–5.0 g/dL
Aspartate transaminase (AST)	38	10–40 U/L
Alanine transaminase (ALT)	75	7–56 U/L
Total bilirubin	1.2	0.1–1.2 mg/dL
Alkaline phosphatase	87	44–147 U/L
Brain natriuretic peptide (BNP)	208	<100 pg/mL
Glomerular filtration rate (GFR)	113	80–120 mL/min/1.73 m^2^
Lactic acid	0.90	0.5–1.6 mmol/L
Urine pH	8	5–9
Nitrite	Negative	Negative
Leukocyte esterase	Negative	Negative
Blood	Negative	Negative
Protein	Negative	Negative
Glucose	Negative	Negative
Urine drug screen (UDS)	Negative	Negative

**Figure 3:**
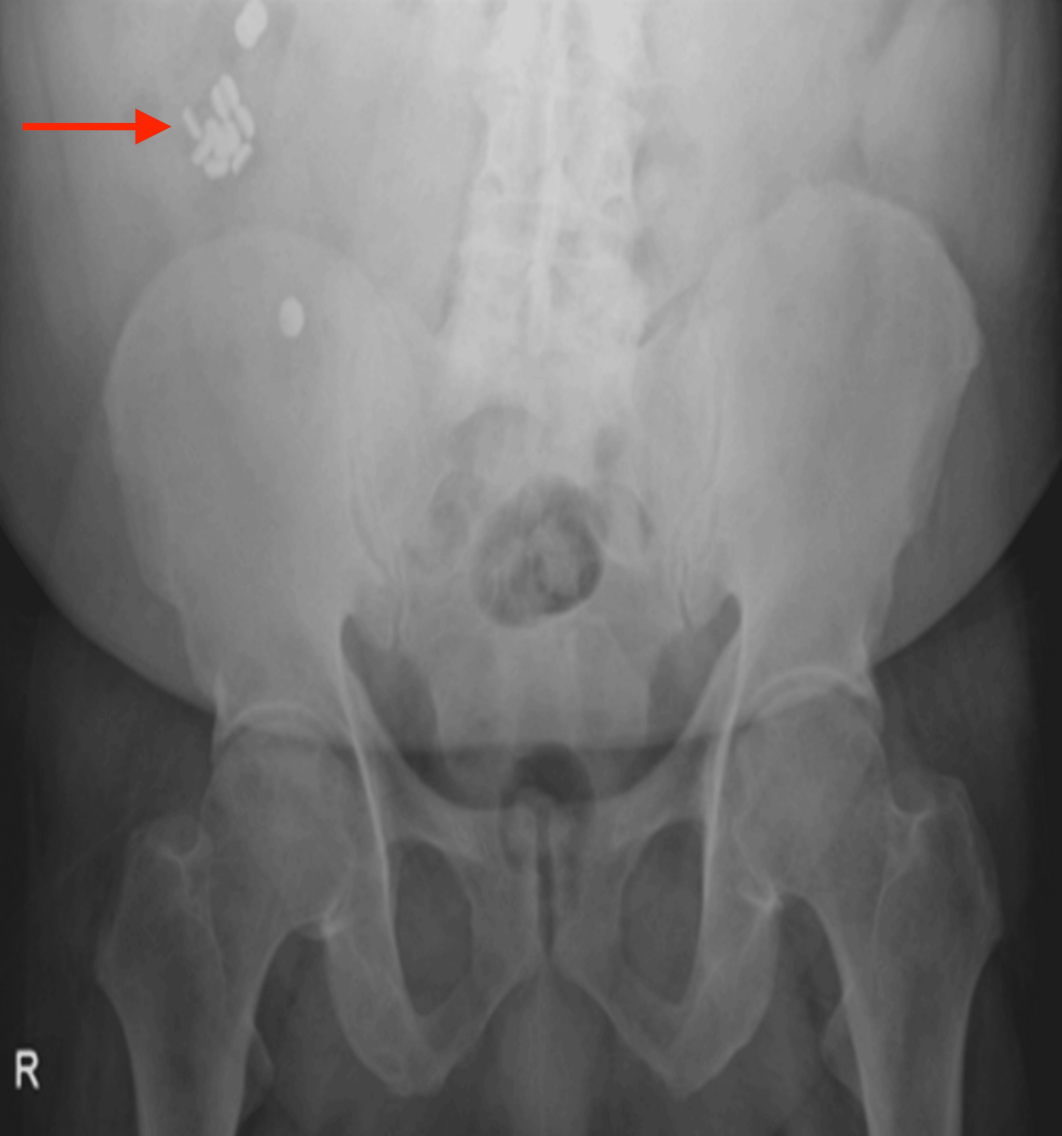
Initial upright abdominal X-ray showing multiple radiopaque foreign bodies seen mainly in the right lower quadrants (red arrow).

**Figure 4:**
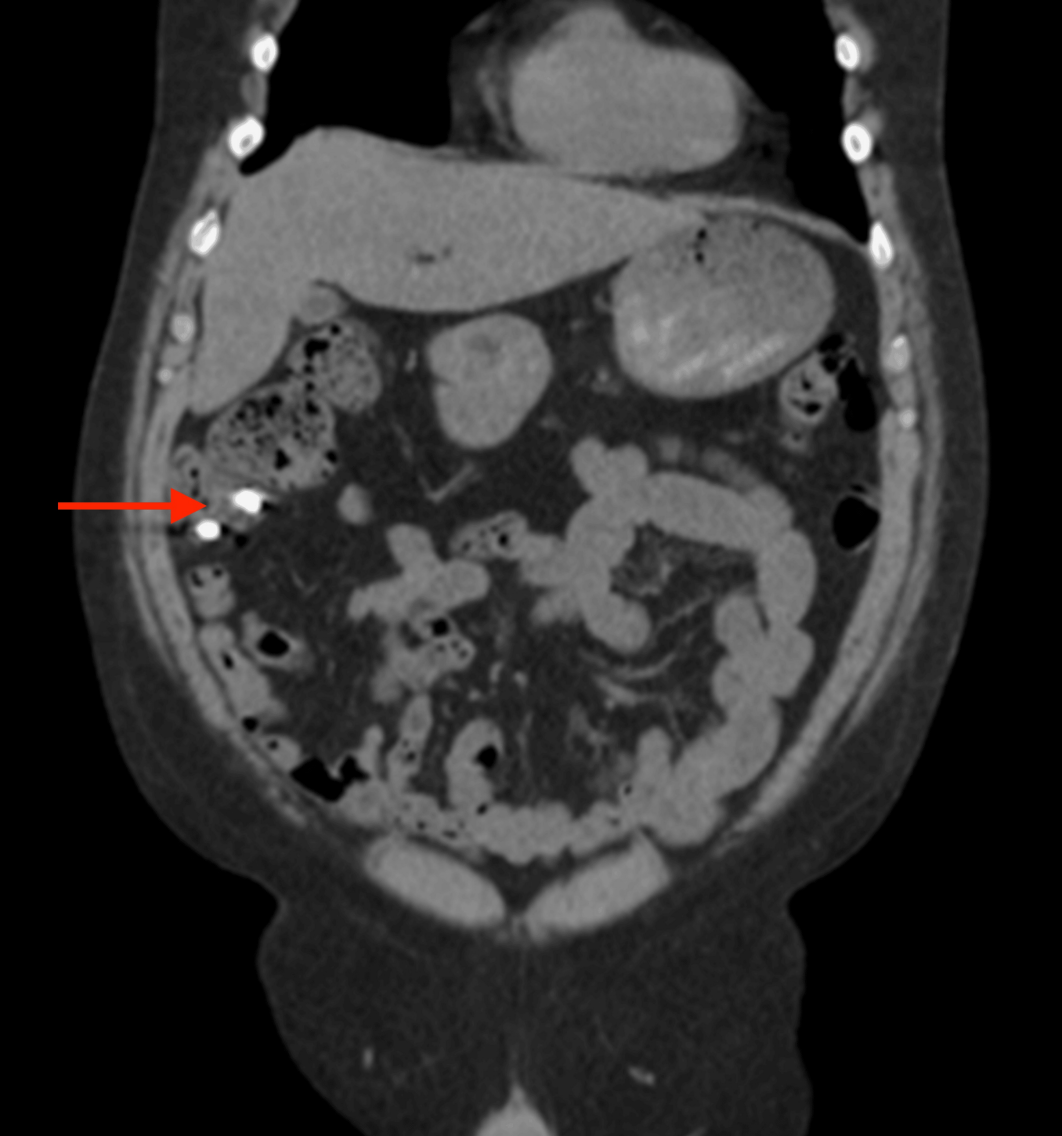
Computed tomography of the abdomen without contrast agents showing multiple sub-centimeter high-density foreign bodies in close approximation with each other within the cecum and ileum (red arrow). No evidence of bowel obstructions or bowel rupture on CT of the abdomen.

The patient was initially treated with 40 mg intravenous furosemide (10 mg/mL) twice daily and 3 mL nebulized albuterol (0.083% 2.5 mg/3 mL) every four hours. With this treatment, his shortness of breath and lower limb swelling began to subside. Despite a partial improvement in symptoms, he was persistently complaining of lower abdominal pain. At this time, the patient noted that he had eaten street food that contained some metal in the past, but further details were not available. Initial abdominal X-ray revealed multiple radiopaque foreign bodies seen primarily within the right lower quadrant and lower mid-abdomen (Figure 3). Subsequently, a computed tomography (CT) of the abdomen without contrast showed multiple sub-centimeter high-density foreign bodies in close approximation with each other within the cecum and ileum (Figure 4). All visualized foreign bodies appear similar in size and shape. There was no evidence of bowel obstruction or bowel rupture on abdominal CT. A multidisciplinary team of internists, gastroenterologists, psychiatrists, and general surgeons was involved in the patient care.

**Figure 5:**
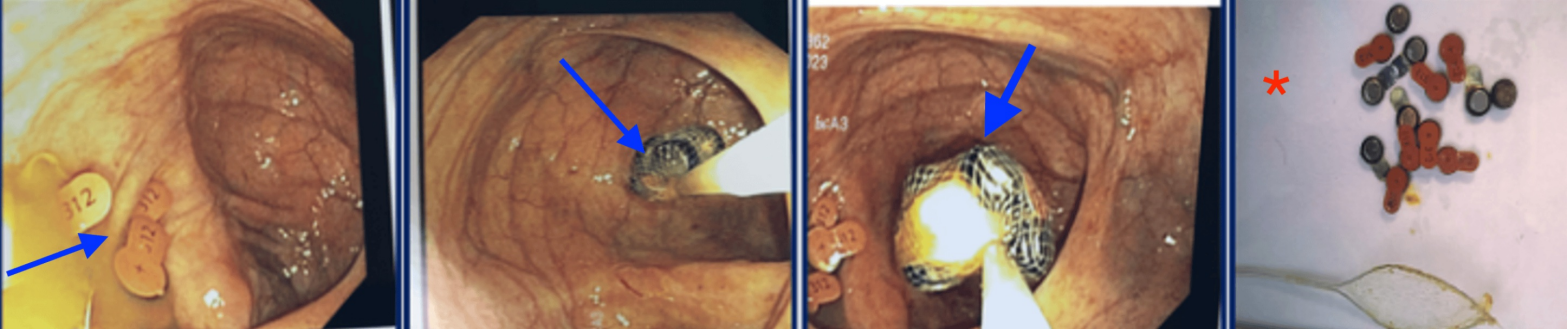
Colonoscopy images showing multiple 312-size zinc and mercury-containing disk batteries (blue arrows). The intraoperative photo of the disc batteries is shown after retrieval (red asterisk).

**Figure 6:**
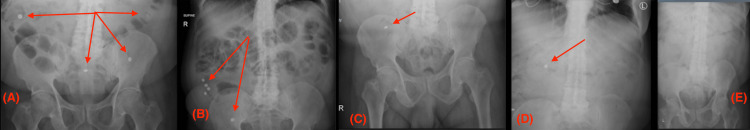
Abdominopelvic X-rays on day two of admission showing the location of batteries in the bowels (A). Colonoscopy was done on day four of admission. Days five to eight are presented in the subsequent X-rays, showing the foreign bodies that were left prior to colonoscopy. (B-D) Subsequent days after colonoscopy showing how foreign bodies are still left (red arrows). At discharge on day eight, no foreign bodies were left (E).

Based on the results of the CT, a colonoscopy was performed, and successful retrieval of fourteen 312-size zinc and mercury-containing disk batteries was achieved (Figure 5). A follow-up abdominopelvic X-ray was done the next day and showed four rounded foreign bodies remaining in the right lower quadrant and two foreign bodies remaining in the right lower pelvis (Figure 6A). Concurrently, the surgical team was on board for a possible exploratory laparotomy. The patient was placed on a bowel regimen, during which serial abdominal X-rays were performed. These X-rays revealed three remaining foreign bodies after two days, and subsequently elimination of all foreign bodies after four days (Figures 6B-6E). The rest of the hospital course remained unremarkable, with continued improvement of abdominal pain. However, given the unusual ingestion of multiple batteries, a psychiatry consultation was performed to assess the patient's mental health status and evaluate any underlying behavioral concerns. The patient exhibited a euthymic mood but reported a history of similar behavioral incidents requiring prior hospital admissions. Despite denying suicidal or homicidal ideation, perceptual disturbances, or delusions, his insight was noted to be impaired. Based on the evaluation, he was diagnosed with recurrent, mild major depressive disorder and initiated on escitalopram for mood stabilization. Emotional support and counseling on medication adherence were provided, and the patient demonstrated understanding of the treatment plan. He remained calm and cooperative throughout his hospital stay, with no further psychiatric decompensation. Prior to discharge, the patient was placed on guideline-directed optimal medical treatment for heart failure with preserved ejection fraction.

## Discussion

Foreign body ingestion in the elderly is a clinically significant yet often overlooked condition, particularly in patients without clear cognitive impairment [7]. While ingestion of button batteries is well-documented in pediatric populations, its occurrence in adults, especially in the absence of intentional ingestion, is rare and presents unique diagnostic and management challenges. This case highlights the importance of maintaining a high index of suspicion for foreign body ingestion in elderly patients presenting with atypical symptoms, particularly when the initial presentation mimics a primary cardiac or pulmonary condition.

Unlike most reported cases, where elderly patients present with dysphagia, choking, or gastrointestinal symptoms following foreign body ingestion, our patient’s chief complaints, i.e., shortness of breath, bilateral lower limb swelling, and abdominal pain, suggested a cardiopulmonary etiology rather than an underlying gastrointestinal issue. This atypical presentation, marked by respiratory and cardiovascular signs rather than the more common gastrointestinal distress, delayed the correct diagnosis, highlighting the need to consider button battery ingestion even when symptoms do not align with classic presentations. The initial clinical suspicion focused on heart failure exacerbation, given the patient's history of coronary atherosclerosis, hypertension, and asthma. It was only through imaging performed for persistent abdominal pain that multiple button batteries were discovered, emphasizing the importance of a thorough assessment to rule out foreign body ingestion in cases with unexplained symptoms.

The comparative analysis with other case reports further illustrates the uniqueness of this case, as seen in Table 2 [7-11]. Most prior reports describe patients presenting with dysphagia or gastrointestinal distress, leading to relatively straightforward endoscopic retrieval of coins, dentures, or small bones. In contrast, our patient unknowingly ingested multiple button batteries, likely hearing aid batteries, which carried an elevated risk of mucosal damage due to their ability to generate an external electric current and release caustic alkaline substances [2]. Furthermore, the potential for systemic toxicity from materials like mercury and zinc in button batteries is a critical consideration. These metals, especially in the event of battery leakage, can lead to severe toxicity, but our patient did not exhibit signs of such complications, which warrants further attention in the management of similar cases.

**Table 2:** Selected studies in the literature presenting with foreign body ingestion in older adults.

Study	Age (years)	Foreign body type	Initial presentation	Imaging methods used	Management	Outcomes
Alturkmani et al. (2023) [7]	67	1 quarter and 1 nickel	Progressive dysphagia	Abdominal X-ray	Endoscopic removal	Resolution of symptoms
Shah & Nemeth (2023) [8]	77	10 coins (4 pennies, 2 dimes, 4 quarters)	Choking, dysphagia	None	Endoscopic removal	Recovery without complications
Marquardt et al. (2020) [9]	88	Dentures	No symptoms exhibited	Abdominal X-ray	Endoscopic retrieval	Successful removal
Nicolas et al. (2017) [11]	73	Chicken bone	Recurrent left lower quadrant pain	Abdominal CT scan	Surgical intervention	Resolution of diverticulitis
Current case	61	14 button batteries	Shortness of breath, lower limb swelling, abdominal pain	Abdominal X-ray, CT scan	Colonoscopy, bowel regimen	Successful elimination of all foreign bodies

Fortunately, despite prolonged retention, the patient avoided major complications such as esophageal perforation, mediastinitis, or systemic toxicity, complications that have been reported in similar cases with delayed intervention [11].

Another critical distinction is the management approach. Unlike other cases where foreign bodies, such as batteries, were removed via esophagogastroduodenoscopy (EGD) soon after presentation, our case required a colonoscopy for retrieval because the batteries had already migrated into the lower gastrointestinal tract. This is significant because it highlights the potential for delayed migration of button batteries, which may require different management approaches, including colonoscopy for retrieval. While colonoscopy for battery retrieval is less commonly reported, it has been documented in select cases involving adult patients, particularly when the foreign body has passed beyond the stomach. However, such instances are rare, and most studies focus on pediatric populations. This required a combination of endoscopic intervention and conservative management with a bowel regimen, which differs significantly from prior reports that often necessitated emergency endoscopy or surgery. The ability of this patient to spontaneously eliminate several batteries over time also raises important questions regarding optimal management strategies in cases where foreign bodies have passed beyond the stomach without immediate complications.

This case emphasizes the importance of thorough history-taking, even when details are limited, and emphasizes the role of early imaging in elderly patients presenting with unexplained gastrointestinal symptoms, particularly when there is persistent discomfort despite treatment for an initially suspected diagnosis. Additionally, this case draws attention to the growing concern of accidental ingestion of household objects, including button batteries, in aging populations, whether due to cognitive decline, polypharmacy-related confusion, or accidental ingestion with food. Finally, this case contributes to our understanding by emphasizing the importance of early detection and individualized management strategies, as well as the need for further research into the risks of toxicity and delayed battery migration in adults.

## Conclusions

This case serves as an important reminder that foreign body ingestion should remain on the differential diagnosis even when patients present with non-specific cardiopulmonary symptoms. Clinicians should maintain a high index of suspicion, particularly in elderly patients, and consider early imaging, such as a chest X-ray or CT scan, when symptoms like dysphagia, persistent cough, or unexplained chest discomfort are present. This is particularly crucial in elderly patients, where delayed recognition can increase morbidity and mortality. Further research is warranted to identify optimal screening strategies for at-risk populations and to refine imaging protocols that could facilitate earlier detection. Additionally, more studies are needed to evaluate alternative management approaches for cases where endoscopic removal is not immediately feasible, such as pharmacologic interventions or novel retrieval techniques.

## References

[1] Vaucel J, Blanc-Brisset I, Tournoud C (2021). Button battery ingestion in older people: prospective study and management algorithm. Toxicol Anal Clin.

[2] Völker J, Völker C, Schendzielorz P (2017). Pathophysiology of esophageal impairment due to button battery ingestion. Int J Pediatr Otorhinolaryngol.

[3] Sethia R, Gibbs H, Jacobs IN, Reilly JS, Rhoades K, Jatana KR (2021). Current management of button battery injuries. Laryngoscope Investig Otolaryngol.

[4] Ettyreddy AR, Georg MW, Chi DH, Gaines BA, Simons JP (2015). Button battery injuries in the pediatric aerodigestive tract. Ear Nose Throat J.

[5] Lee JH (2018). Foreign body ingestion in children. Clin Endosc.

[6] Chiew AL, Chan BS (2023). Management of button battery ingestion. Clin Toxicol (Phila).

[7] Alturkmani OG, Al-Badawi MM, Alturkmani SG, Al-Midani MH, Attar SA (2023). A case report of non-intentional foreign body ingestion in an elderly patient. Cureus.

[8] Shah S, Nemeth A (2023). Foreign body ingestion: an unusual case in a patient with dementia. Cureus.

[9] Marquardt P, Derousseau T, Patel N (2020). Foreign body ingestion: a curious case of the missing denture. Geriatrics (Basel).

[10] Libânio D, Garrido M, Jácome F, Dinis-Ribeiro M, Pedroto I, Marcos-Pinto R (2018). Foreign body ingestion and food impaction in adults: better to scope than to wait. United European Gastroenterol J.

[11] Nicolas GN, Assaker R, Saliba C (2017). Foreign body ingestion causing recurrent diverticulitis. Am J Case Rep.

